# Efficacy of postoperative seizure prophylaxis in intra-axial brain tumor resections

**DOI:** 10.1007/s11060-014-1402-9

**Published:** 2014-02-17

**Authors:** Shaheryar F. Ansari, Bradley N. Bohnstedt, Susan M. Perkins, Sandra K. Althouse, James C. Miller

**Affiliations:** 1 Department of Neurological Surgery, Goodman Campbell Brain and Spine, Indiana University, 355 W 16th St. Ste 5100, Indianapolis, IN 46202 USA; 2 Department of Biostatistics, Indiana University School of Medicine, Indianapolis, IN USA

**Keywords:** Anticonvulsants, Brain neoplasms, Seizure, Postoperative complications, Neurosurgery

## Abstract

The effectiveness of seizure prophylaxis in controlling postoperative seizures following craniotomy for tumor resection is unclear. Most patients are seizure-free before surgery. To prevent seizures, it is common to treat tumor craniotomy patients postoperatively with an antiepileptic drug (AED). The authors retrospectively analyzed seizure occurrence with and without postoperative prophylactic AEDs. Between 2005 and 2011 at the authors’ institution, 588 patients underwent craniotomy for brain tumors and were screened. Data on seizures, AED use, histopathology, comorbidities, complications, and follow-up were collected. Exclusion criteria included lack of follow-up data, previous operation, preoperative seizures, or preoperative AED prophylaxis. The incidence of postoperative seizures in patients with and without prophylactic AEDs was compared using logistic regression analysis. A total of 202 patients (50.5 % female) were included. The most common tumor diagnosis was metastasis (42.6 %). Of the 202 patients, 66.3 % were prescribed prophylactic AED after surgery. Forty-six of 202 (22.8 %) suffered a postoperative seizure. The odds of seizure for patients on prophylactic AED was 1.62 times higher than those not on AED (*p* = 0.2867). No difference was found in seizure occurrence between patients with glioblastoma multiforme compared with other tumor types (odds ratio 1.75, *p* = 0.1468). No difference was found in time-to-seizure between the two groups (hazard ratio 1.38, *p* = 0.3776). These data show no statistically significant benefit to prophylactic postoperative AED and a nonsignificant trend for increased seizure risk with AEDs. A randomized, placebo-controlled trial is needed to clarify the benefit of postoperative AED use for brain tumor resection.

## Introduction

Patients undergoing surgery for brain tumors have an estimated incidence of seizures ranging from 17 to 50 % [1–6]. Studies that have investigated the question whether seizure prophylaxis benefits such patients have shown mixed results. Although the double-blinded, randomized controlled trial of North et al. [7] found a significant reduction of postoperative seizures in a specific time period, other studies did not corroborate this finding [6, 8, 9]. On the other hand, a relatively recent retrospective review by Zachenhofer et al. [5] found that patients given prophylactic levetiracetam may have a lower risk of seizure following craniotomy for tumor.

Most of the studies published on postoperative seizure prophylaxis included heterogeneous populations—brain tumors, aneurysms, or trauma. The studies that focused on brain tumors included both intra- and extra-axial tumors, and since most of them are older, they used older antiepileptic drugs (AEDs). Newer medications have emerged that are now widely used.

A recent randomized controlled trial by Wu et al. [2] did not demonstrate a significant difference in postoperative seizure occurrence between patents who had been given prophylactic medication (specifically, phenytoin) and those who had not. Notably, this trial was canceled for insufficient power to detect a significant difference. A similar trial that was attempted several years earlier by DeSantis et al. [10] was also abandoned because of insufficient power, but this study included patients with extra-axial lesions.

Before the study by Wu et al. [2], no study had specifically focused on the efficacy of postoperative seizure prophylaxis in patients with intrinsic brain tumors. To establish the efficacy of postoperative seizure prophylaxis in this population, we conducted a retrospective study of intra-axial brain tumor operations performed at our institution from 2005 to 2011. The ultimate question we seek to answer is whether the potential benefit of giving prophylactic AEDs after brain tumor surgery outweighs the risk of adverse drug effects and the associated costs.

## Materials and methods

All records of patients who underwent craniotomy for intra-axial tumor resection in our institution between 2005 and 2011 were retrospectively reviewed. The records were scanned for specific data: sex, age at diagnosis, tumor pathology, occurrence of seizures before surgery, use of an AED before surgery, use of polifeprosan/carmustine wafers (Gliadel™) in the cases of glioblastoma multiforme (GBM) patients, use of seizure prophylaxis after surgery, and the occurrence of seizures before or after surgery. The initial review resulted in a database of 588 patients. Sixteen patients were then excluded because of missing charts and misclassification of surgery.

Included patients were over 18 years old without preoperative seizures or preoperative AED use. Only first operations were considered. Information on specific complications of AED therapy and characteristics of seizure disorders, such as semiology and frequency, were incomplete and often not available. The AED medications used included phenytoin (26 %), levetiracetam (63 %), and others, including carbamazepine, phenobarbital, topiramate, and valproate (combined, 11 %). No specific method or algorithm was used to determine which patients were placed on postoperative AEDs—rather, individual physician preference was followed with regard to which patients received prophylaxis.

Patients who had undergone surgery before 2005 or at an outside institution were excluded. Patients with insufficient data available in the medical record were also excluded. Such patients were those with missing records, patients whose records had no information about preoperative seizure status, and patients who had no follow-up documented in our records.

This resulted in 266 patients, and 202 of these patients had seizure outcome data available. The 64 patients with no seizure outcome data available were not significantly different from the 202 with respect to sex and tumor pathology, but those without information regarding seizures were, on average, older than those who had these data (mean ± SD 61 ± 12 vs. 56 ± 15 years). We analyzed the 202 patients with available seizure outcome data with regard to whether they were given prophylactic AEDs and whether they experienced a seizure postoperatively.

We tested whether prophylactic AED use was associated with seizure occurrence with logistic regression, adjusting for correlation due to within-attending physician variations using generalized estimating equations methodology. Within-attending physician correlation can occur because of physician preferences and habits. Our null hypothesis was that the odds of having a seizure postoperatively are the same whether or not a patient is placed on a prophylactic AED.

Due to the large differences in follow-up among patients, we also conducted a time-to-seizure analysis using extended Cox proportional hazards regression models using frailty modeling. The time to event was calculated as time from surgery until seizure. The null hypothesis was that the time-to-seizure curve was the same for the prophylaxis group and the no-prophylaxis group. If a patient did not have any seizures recorded, their time-to-seizure value was censored and determined by calculating the time from surgery until the last recorded follow-up time. Time-to-seizure curves were estimated using the Kaplan–Meier method. Similar models as described above were fit to compare GBM to other pathologies and to assess the effect of AEDs for GBM patients only, as well as for the GBM subset analysis with Gliadel. The latter models started with the main effects for prophylaxis and Gliadel use and the interaction of these two effects. If the interaction term was not significant, it was removed from the model. All analyses were performed using SAS for Windows version 9.3.

## Results

Demographics for our patient population are shown in Table 1. The population was 50.5 % (102 of 202) female with a median age at diagnosis of 55.5 years (range 20–83). Table 2 summarizes follow-up and time to seizure after surgery and also shows that 46 of 202 (22.8 %) suffered a recorded seizure postoperatively. A total of 134 (66.3 %) of the 202 patients were prescribed prophylactic AED postoperatively, with older patients more likely to be given AEDs (Table 3). Also in the data shown in Table 3, no significant difference in AED usage was found with different tumor locations.

**Table 1:** Demographic information, all patients

Variable	Number (%) or Median (range)
Sex	
Female	102 (50.50)
Male	100 (49.50)
Age at diagnosis (years)	55.5 (20–83)
Tumor pathology	
Colloid cyst	1 (0.50)
GBM	74 (36.63)
Metastasis	86 (42.57)
Non-GBM glioma	28 (13.86)
Other	13 (6.44)
Gliadel use (in GBM patients)	
No	51 (85.00)
Yes	9 (15.00)

**Table 2:** Seizure occurrence and follow-up

Variable	Number (%) or Median (Range)
Follow-up time (days)	321 (6–4,882)
Time from surgery to seizure (days)	205 (3–2,281)
Postsurgery seizure	
No	156 (77.2 %)
Yes	46 (22.8 %)
Postsurgery AED use	
No	68 (33.66 %)
Yes	134 (66.34 %)

**Table 3:** Univariate analyses of postoperative AED use

Variable	Number of patients with AEDs prescribed/total	Odds Ratio (95 % CI)	*p* Value (Type 3 analysis of effects from Proc Logistic)
Sex			0.6205
Female^a^	66 of 102		
Male	68 of 100	1.159 (0.646, 2.079)	
Age at diagnosis (years)		0.973 (0.953, 0.993)	0.0095
Tumor pathology			0.9277
Colliod cyst	1 of 1	–	
GBM^a^	47 of 74		
Metastasis	53 of 86	0.923 (0.485, 1.754)	
Non-GBM glioma	20 of 28	1.436 (0.557, 3.701)	
Other	13 of 13	–	
Gliadel use (in GBM patients)			0.6008
No^a^	33 of 51		
Yes	5 of 9	0.682 (0.162, 2.863)	
Side			0.6928
Left^a^	67 of 103		
Right	67 of 99	1.125 (0.627, 2.018)	
Lobe			0.8080
Parietal	36 of 52	1.313 (0.619, 2.781)	
Ventricle	6 of 8	1.750 (0.330, 9.267)	
Insular	1 of 1	–	
Temporal	34 of 48	1.417 (0.651, 3.083)	
Frontal^a^	48 of 76		
Occipital	8 of 14	0.778 (0.245, 2.473)	
Basal Ganglia	1 of 3	0.292 (0.025, 3.364)	

^a^Reference category

As shown in Table 4, we found no difference in seizure occurrence when we compared patients with GBM with those with other tumor pathologies (*p* = 0.6257). Twenty-one of 74 (28.4 %) patients with GBM had seizures, and the odds of having a seizure postoperatively was 1.75 times higher for patients who were on a prophylactic AED than not (*p* = 0.3300). Sixty of the 74 patients (81.1 %) with GBM had information recorded regarding the use of Gliadel, and 9 of these (15 %) had been given Gliadel. The seizure rates of these patients are summarized in Table 4. Tumor location and side were not significantly associated with seizure occurrence. Analysis was attempted to find a difference between early (within 30 days) and late postoperative seizures, however only two patients in this series were found to have seizures within 30 days of surgery, and neither patient was on AED prophylaxis.

**Table 4:** Univariate analyses of patients who experienced seizure after surgery, all patients

Variable	Number of patients with seizure/total	Odds Ratio (95 % CI)	*p* Value (Type 3 analysis of effects from Proc Logistic)
Sex			0.9391
Female^a^	23 of 102		
Male	23 of 100	1.026 (0.532, 1.980)	
Age at diagnosis (years)		0.995 (0.973, 1.016)	0.6196
Tumor pathology			0.6257
Colliod cyst	0 of 1	–	
GBM^a^	21 of 74		
Metastasis	16 of 86	0.577 (0.275, 1.211)	
Non-GBM glioma	7 of 28	0.841 (0.311, 2.272)	
Other	2 of 13	0.459 (0.094, 2.248)	
Gliadel use (in GBM patients)			0.6250
No^a^	13 of 51		
Yes	3 of 9	1.462 (0.319, 6.689)	
Side			0.4106
Left^a^	21 of 103		
Right	25 of 99	1.319 (0.682, 2.552)	
Lobe			0.9590
Parietal	9 of 52	0.674 (0.276, 1.646)	
Ventricle	2 of 8	1.074 (0.199, 5.794)	
Insular	0 of 1	–	
Temporal	12 of 48	1.074 (0.463, 2.489)	
Frontal^a^	18 of 76		
Occipital	4 of 14	1.289 (0.360, 4.610)	
Basal Ganglia	1 of 3	1.611 (0.138, 18.820)	

^a^Reference category

**Fig. 1:**
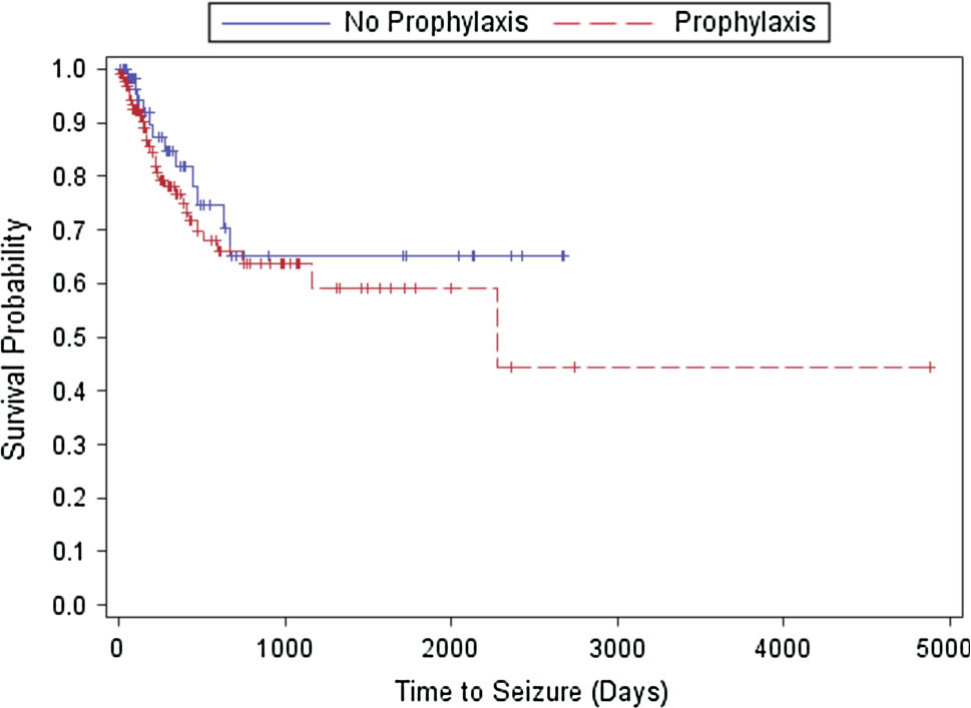
Kaplan–Meier time-to-seizure curves

Twelve of 68 (17.6 %) of those who had not been given a prophylactic AED, and 34 of 134 (25.4 %) who had received a prophylactic AED [odds ratio (OR) 1.62, *p* value = 0.2867] had seizures. Similarly, the hazard ratio in the time-to-event model was 1.38 (*p* value = 0.3776), indicating higher risk for seizure in the group receiving prophylactic AEDs; however, as with the odds ratio, the effect was not significant. The Kaplan–Meier curve is shown in Fig. 1. Adjusting for age had no substantive impact on the estimates or p values. In both logistic and time-to-event models, there was no interaction between prophylactic AED and Gliadel use on postoperative seizures, and the main effect for Gliadel use was not significant when the interaction term was removed from the models.

## Discussion

The results of this study based on our data set suggest that there may not be a difference in seizure occurrence with the use of prophylactic AED. This is in agreement with the findings from most studies in the literature on this topic [2–4, 6, 9–14]. The randomized controlled trial by Wu et al. [2] was discontinued because the investigators found that the incidence of seizures was lower than expected based on preliminary assumptions. In that study, the overall incidence of early postoperative seizures was 8 %, as opposed to the expected incidence of 30 %. The study was thus underpowered and would have required more than 700 patients to detect a significant difference between the groups. This may suggest that the rate of postoperative seizures in brain tumor patients may have decreased over time from the historical rates of 15–20 % [1, 6, 7, 15, 16]. However, a closer look at the data in the Wu [2] study shows that the overall incidence of seizures (early and late) was approximately 21 %. In this study, the rate of postoperative seizures was 22.8 % overall, which is very similar to the published incidence in other groups. The incidence of postoperative seizures in the published reports varies from 17 to 50 %, with a trend toward lower rates in recent years [1–6]. In our study, we did not differentiate between early and late seizures, as only two patients had seizures prior to 30 days, neither of whom was given an AED. This is suggestive of a more general statement, supported by previous literature [5, 6], that the modern perioperative (less than 30 days after surgery) seizure rate is in fact very low. The only statistically significant finding in our analysis was that older patients were more likely to receive AED prophylaxis. This may reflect a greater degree of caution toward this older population, or it could be a presumption of higher risk of postoperative seizure or greater morbidity should a seizure occur. Interestingly, an analysis stratified by tumor location within the brain did not find a statistically significant difference in seizure occurrence or AED usage with tumors e.g. in the temporal lobe—a location which traditionally is considered to be at a higher risk for seizures.

It was believed that placement of Gliadel wafers may cause more swelling and irritation of the surrounding cortex, thus predisposing these patients to postoperative seizures. However, patients who had Gliadel did not seem to have a higher rate of seizure occurrence than their counterparts without Gliadel. The sample size in this case is small. It also was not possible to control for which patients received the wafers—this was based on attending physician judgment regarding whether their use was merited in a given patient. The subset as a whole showed the same lack of efficacy of prophylaxis as the rest of the population.

Most prior studies were performed using older AEDs, usually phenytoin, which is associated with many potentially dangerous side effects—the overall adverse event rate for phenytoin is estimated to be approximately 15 % [11, 17]. Furthermore, phenytoin has known effects on the metabolism of other medications through the CYP enzyme system. This has significant implications for patients receiving adjuvant therapy [5, 13, 18]. Levetiracetam has a relatively more benign side effect profile [5]; side effect frequency is estimated to be from 5 to 27 % [5, 19]. Levetiracetam is not known to induce the CYP system and thus interact with other drugs. These characteristics make levetiracetam a more attractive prophylactic agent, and indeed our institution uses levetiracetam almost exclusively in patients undergoing craniotomy. However, levetiracetam is associated with higher costs to the patient, and to date no randomized controlled trial has been performed demonstrating its efficacy in seizure prevention.

## Limitations

This study is retrospective, which limits its ability to draw strong conclusions. Furthermore, we were able to work only with the information available in the medical records, which at times was limited and resulted in the exclusion of a large number of patients. This creates a significant limitation in the power of the study to actually find a difference, if one is present. This lack of power is particularly more prominent when the population is broken down into subgroups, as done here. There may also be a selection bias regarding which patients were selected for prophylactic AED. Indeed, the fact that the prophylaxis group had a nonsignificantly higher rate of seizure occurrence could indicate these patients had a higher presumed risk of developing postoperative seizures. This is controllable only in a prospective, randomized controlled setting. Also, late-onset seizures may be the presenting symptoms of a recurrence and implies a potentially different pathophysiology than an isolated seizure postoperatively, and in a prospective trial, patients with recurrence should ideally be excluded from the analysis.

In conclusion, this single-institution retrospective analysis of postoperative seizure prophylaxis in patients undergoing surgery for intra-axial brain tumors did not find a statistically significant difference in the occurrence of seizures between prophylaxed and non-prophylaxed patients—this is not to imply that no difference is present when this population is considered as a whole, but the analysis of the data from our institution does not seem to indicate a statistical difference. This result is supported by the majority of recent studies. Based on data from our study and others, we believe the use of AED prophylaxis for patients undergoing craniotomy for intrinsic brain tumor resection is a treatment option, but it may not need to be used as an automatic treatment for all patients. However, further work remains in this area. The overall seizure incidence in this population was 22.8 %, which falls nicely into the range frequently cited in the literature. The seeming clinical equipoise in this area suggests that a large, multicenter, prospective, randomized controlled study of levetiracetam prophylaxis after craniotomy for intrinsic brain tumor should be conducted. Further studies should include information about seizure frequency in the study population, and also about side effects and complications of therapy, as well as treatment costs to allow for determination of a risk/benefit ratio. This would serve to provide meaningful information about benefits and cost-effectiveness for postoperative seizure prophylaxis in craniotomy for intrinsic brain tumor surgery.

## References

[1] Foy PM, Copeland GP, Shaw MD (1981). The incidence of postoperative seizures. Acta Neurochir.

[2] Wu AS, Trinh VT, Suki D (2013). A prospective randomized trial of perioperative seizure prophylaxis in patients with intraparenchymal brain tumors. J Neurosurg.

[3] Glantz MJ, Cole BF, Fosyth PA (2000). Practice parameter: anticonvulsant prophylaxis in patients with newly diagnosed brain tumors: report of the Quality Standards Subcommittee of the American Academy of Neurology. Neurology.

[4] Cohen N, Strauss G, Lew R, Silver D, Recht L (1988). Should prophylactic anticonvulsants be administered to patients with newly-diagnosed cerebral metastases? A retrospective analysis. J Clin Oncol.

[5] Zachenhofer I, Donat M, Oberndorfer S, Roessler K (2011). Perioperative levetiracetam for prevention of seizures in supratentorial brain tumor surgery. J Neurooncol.

[6] Shaw MD, Foy P, Chadwick D (1983). The effectiveness of prophylactic anticonvulsants following neurosurgery. Acta Neurochir.

[7] North JB, Penhall RK, Hanieh A, Frewin DB, Taylor WB (1983). Phenytoin and postoperative epilepsy. J Neurosurg.

[8] Kuiljen JM, Teernstra OP, Kessels AG, Herpers MJ, Beuls EA (1996). Effectiveness of antiepileptic prophylaxis used with supratentorial craniotomies: a meta-analysis. Seizure.

[9] Foy PM, Chadwick DW, Rajgopalan N, Johnson AL, Shaw MD (1992). Do prophylactic anticonvulsant drugs alter the pattern of seizures after craniotomy?. J Neurol Neurosurg Psychiatry.

[10] De Santis A, Villani R, Sinisi M, Stocchetti N, Perucca E (2002). Add-on phenytoin fails to prevent early seizures after surgery for supratentorial brain tumors: a randomized controlled study. Epilepsia.

[11] Deutschmann C, Haines SJ (1985). Anticonvulsant prophylaxis in neurological Surgery. Neurosurgery.

[12] Hayashi T, Hadeishi H, Kawamura S, Nonoyama Y, Suzuki A, Yasui N (1999). Postoperative anticonvulsant prophylaxis for patients treated for cerebral aneurysms. Neurol Med Chir.

[13] Sirven JI, Wingerchuk DM, Drazkowski JF, Lyons MK, Zimmerman RS (2004). Seizure prophylaxis in patients with brain tumors: a meta-analysis. Mayo Clin Proc.

[14] Young B, Rapp RP, Norton JA, Haack D, Tibbs PA, Bean JR (1983). Failure of prophylactically administered phenytoin to prevent early posttraumatic seizures. J Neurosurg.

[15] Sbieh I, Tamas LB, O’Laoire SA (1986). Epilepsy after operation for aneurysms. Neurosurgery.

[16] Kvam D, Loftus CM, Copeland B, Quest DO (1983). Seizures during the immediate postoperative period. Neurosurgery.

[17] Moots PL, Maciunas RJ, Eisert DR, Parker RA, Laporte K, Abou-Khalil B (1995). The course of seizure disorders in patients with malignant gliomas. Arch Neurol.

[18] Hildebrand J, Lecaille C, Perennes J, Delattre J-Y (2005). Epileptic seizures during follow-up of patients treated for primary brain tumors. Neurology.

[19] Goldberg-Stern H, Feldman L, Eidlitz-Markus T (2013). Levetiracetam in children, adolescents and young adults with intractable epilepsy: efficacy, tolerability and effect on electroencephalogram—a pilot study. Eur J Paediatr Neurol.

